# Alarming attrition rates among HIV-infected individuals in pre-antiretroviral therapy care in Myanmar, 2011–2014

**DOI:** 10.3402/gha.v9.31280

**Published:** 2016-08-24

**Authors:** Myo Minn Oo, Vivek Gupta, Thet Ko Aung, Nang Thu Thu Kyaw, Htun Nyunt Oo, Ajay MV Kumar

**Affiliations:** 1 International Union Against Tuberculosis and Lung Disease, Mandalay, Myanmar; 2 International Union Against Tuberculosis and Lung Disease, South-East Asia Regional Office, New Delhi, India; 3 International Union Against Tuberculosis and Lung Disease, Paris, France; 4 Community Ophthalmology, Dr. RP Centre for Ophthalmic Sciences, All India Institute of Medical Sciences, New Delhi, India; 5 National AIDS Program, Ministry of Health, Myanmar

**Keywords:** pre-ART care, retention, lost to follow-up, death, SORT IT, operational research

## Abstract

**Background:**

High retention rates have been documented among patients receiving antiretroviral therapy (ART) in Myanmar. However, there is no information on human immunodeficiency virus (HIV)-infected individuals in care before initiation of ART (pre-ART care). We assessed attrition (loss-to-follow-up [LTFU] and death) rates among HIV-infected individuals in pre-ART care and their associated factors over a 4-year period.

**Design:**

In this retrospective cohort study, we extracted routinely collected data of HIV-infected adults (>15 years old) entering pre-ART care (June 2011**–**June 2014) as part of an Integrated HIV Care (IHC) programme, Myanmar. Attrition rates per 100 person-years and cumulative incidence of attrition were calculated. Factors associated with attrition were examined by calculating hazard ratios (HRs).

**Results:**

Of 18,037 HIV-infected adults enrolled in the IHC programme, 11,464 (63%) entered pre-ART care (60% men, mean age 37 years, median cluster of differentiation 4 (CD4) cell count 160 cells/µL). Of the 11,464 eligible participants, 3,712 (32%) underwent attrition of which 43% were due to deaths and 57% were due to LTFU. The attrition rate was 78 per 100 person-years (95% CI, 75**–**80). The cumulative incidence of attrition was 70% at the end of a 4-year follow-up, of which nearly 90% occurred in the first 6 months. Male sex (HR 1.5, 95% CI 1.4–1.6), WHO clinical Stage 3 and 4, CD4 count <200 cells/µL, abnormal BMI, and anaemia were statistically significant predictors of attrition.

**Conclusions:**

Pre-ART care attrition among persons living with HIV in Myanmar was alarmingly high – with most attrition occurring within the first 6 months. Strategies aimed at improving early HIV diagnosis and initiation of ART are needed. Suggestions include comprehensive nutrition support and intensified monitoring to prevent pre-ART care attrition by tracking patients who do not return for pre-ART care appointments. It is high time that Myanmar moves towards a ‘test and treat’ approach and ultimately eliminates the need for pre-ART care.

## Introduction

Globally, human immunodeficiency virus (HIV) infection continues to be an important infectious disease causing mortality and morbidity, particularly in developing countries. The burden of HIV in the Asia-Pacific region is high, with an estimated 4.9 million people living with HIV (PLHIV) (1). The number of PLHIV in Myanmar in 2013 was approximately 189,000 (2). Although HIV prevalence in Myanmar declined from 0.6% in 2010 to 0.5% in 2013, the burden in terms of absolute numbers remains high. In 2013, about 15,000 people died of AIDS-related causes and there were 7,000 new infections (1).

In 2011, Myanmar had about 90,000 patients who needed antiretroviral therapy (ART) (based on cluster of differentiation 4 CD4 cell counts less than 200 cells/µL), and 40,128 (44%) of them were receiving ART. Access to ART care in the country increased in 2012 with the National AIDS Programme (NAP) adopting the revised World Health Organization-2010 (WHO) recommendations of increasing the CD4 threshold for ART initiation to 350 cell/mm^3^ (2, 3). These recommendations increased the needs for ART care in Myanmar to 125,333 patients. Although overall ART coverage was 43%, 53,709 new patients commenced ART as a result of the change in recommendations. Once treatment started, these patients were actively followed up by the health system, and retention rates were fairly high (4). A study from Myanmar published in 2014 reported that nearly three-quarters of the HIV-infected patients receiving ART under the Integrated HIV Care (IHC) programme were still in care after 5 years (5).

Although retention is high among patients who have started ART, there is no information about the retention of those in pre-ART care, that is, care before initiation of ART. Studies from India and Africa show that pre-ART care attrition could be as high as 40% at 1 year after registration (5–8). Anecdotal evidence suggests that this could be similar in Myanmar, although prior to this study there was no clear evidence of this.

Myanmar is currently developing National Strategic Plans with the aim of achieving 90-90-90 goals related to HIV (diagnosing 90% of all PLHIV, treating 90% of those diagnosed, and achieving viral suppression in 90% of those treated) and finally ending the HIV/AIDS pandemic in line with the United Nations’ Sustainable Development Goals by 2030 (9). Hence, it is important to understand the outcomes among PLHIV in pre-ART care. Thus, among PLHIV enrolled in pre-ART care during 2011–2015 as part of the IHC programme in Myanmar, we aimed to assess attrition rates (loss-to-follow-up [LTFU] and death) and the socio-demographic and clinical factors associated with attrition.

## Methods

### Ethics

The Ethics Advisory Group of the International Union Against Tuberculosis and Lung Disease (The Union), Paris, France, approved the study and waived the need for individual informed consent because the study involved a review of existing programme records. The NAP of Myanmar permitted us to use the data for publication. To ensure confidentiality, we excluded patient identifiers from the final database prior to analysis.

### Study design

This is a retrospective cohort study using routinely collected data.

### Study setting

The IHC programme is being implemented within the public sector by The Union, in collaboration with the Department of Health, National Tuberculosis (TB) Programme (NTP) and NAP since 2005 in Myanmar. Initial implementation was at a single ART clinic in Mandalay. Since then, there has been a steady expansion of the programme, and currently, ART is delivered through 18 ART centres and 14 ART decentralized sites. By the end of 2014, more than 20,000 patients were receiving ART in the IHC programme running across the country. Every month, around 400 patients are enrolled in the programme. Patients with a confirmed HIV diagnosis are referred from hospitals and health centres, STD (sexually transmitted disease)/HIV-AIDS clinics, TB clinics, antenatal clinics and ART programmes run by other organizations apart from the IHC.

**Fig. 1:**
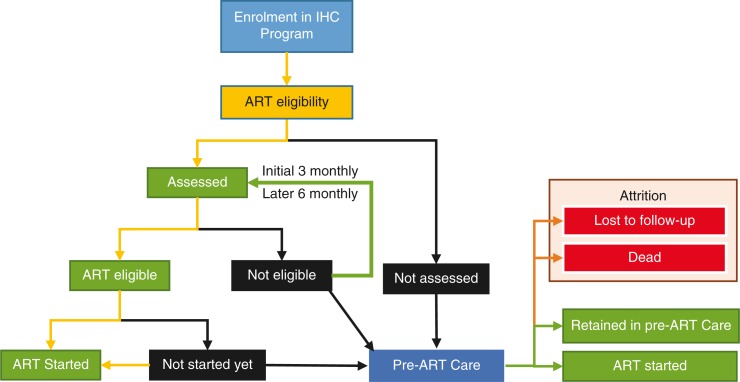
Patient flow and assessment procedures for HIV-infected individuals after enrolment in IHC programme, Myanmar. IHC, Integrated HIV Care; ART, antiretroviral therapy.

Patient flow and assessment procedures after enrolment in IHC programme as well as the potential points of attrition in pre-ART care are shown in Fig. 1. Details of follow-up care of patients on ART have been previously published (5). After the patients are enrolled, they receive ART if they are eligible based on clinical or immunological criteria. Eligibility criteria for ART initiation are WHO clinical staging 3 or 4, and/or CD4 counts less than the threshold (200 cells/µL before 2012 and 350 cells/µL after 2012) (4, 10). For all enrolled patients, a second visit is needed to assess the results of CD4 count and other laboratory investigations for completion for ART eligibility evaluation. If patients do not come for a second visit within 6 weeks of enrolment, they are classified as ‘not assessed for eligibility at enrolment’. If the patient's baseline CD4 is higher than the threshold or did not fall under WHO clinical Stage 3 or 4, she/he is categorized at the second visit as ‘not eligible for ART at enrolment’. There are patients who are eligible but are not initiated on ART within 6 weeks due to other reasons (deaths, LTFU due to social or economic reasons and patient refusal to start ART). These patients are categorized as ‘not started on ART at enrolment’. As shown in Fig. 1, PLHIV not assessed for eligibility, not eligible for ART, and not started on ART at enrolment are operationally defined here as ‘patients under the pre-ART care’. They are the patients who needed to be followed up for either completion of assessment, reassessment for eligibility, or for the earliest possible initiation of ART, respectively.

The PLHIV who are assessed and are not yet eligible for ART are usually clinically healthy and receive multivitamins, drugs for treatment of opportunistic infections, and counselling for psychosocial support. These patients are followed up after 3 months when ART eligibility is reassessed using the same clinical and immunological criteria described above. Subsequent follow-up visits of patients in pre-ART care usually occur at 6 monthly intervals but may be more frequent as per the discretion of the treating physician and as per patient needs.

The PLHIV who are eligible for ART but have not yet started on treatment are usually ill and hence prioritized for receiving the next appointment in 1 or 2 weeks’ time. Patients who are not assessed for eligibility at enrolment are also scheduled for next appointment depending on their clinical condition. All of these services are provided free of cost to the patients.

The PLHIV taking ART in IHC programme are also involved as volunteers, actively participating in clinic activities such as triage, registration, adherence counselling, and psychosocial support, and they play an important role in tracing patients who are lost to follow-up. Most of these efforts are targeted towards those already on ART and incorrect addresses pose major problems. Reasons include a migratory population, fear of discrimination, and long travel distances to receive care.

Every patient has a dedicated file in which clinical details are entered by the treating doctors in IHC clinics. Data in these records are electronically captured in ‘IUATLD epi-concept’ database on a daily basis by trained data entry operators and medical doctors. Data quality control is performed at regular intervals to minimize errors in data entry and ensure data validity and consistency.

### Study population

We included all HIV-infected adults (>15 years of age) enrolled into pre-ART care of IHC programme, Myanmar, between 1^st^ June 2011 and 30^th^ June 2014. We excluded patients aged ≤15 years and women under a prevention of mother-to-child transmission (PMTCT) programme because of the different follow-up and clinical management schedule. Patients transferred out to other ART facilities were also excluded.

### Data collection

Data variables for demographic, clinical, and immunological characteristics included age, sex, marital status, literacy, employment, baseline WHO clinical staging, CD4 count, body mass index (BMI: defined as body weight in kilograms divided by the square of their height in meter [kg/m^2^]), haemoglobin level, and dates of enrolment, ART initiation, LTFU, last appointment, and hepatitis B and C infection status at enrolment. Based on haemoglobin (grams per decilitre) level, we divided the patients into four categories: normal (male, ≥13; female, ≥12), mild (male, 11–12.9; female, 11–11.9), moderate (both sexes, 8–10.9), and severe anaemia (both sexes, <8) (11). Data for these variables were extracted from the ‘IUATLD epi-concept’ database and exported for analysis. Patient records were anonymized and de-identified prior to analysis.

Outcomes of HIV-infected adults who entered pre-ART care in IHC programme are shown in Fig. 1. There were four operationally defined outcomes: 1) ‘Retained in pre-ART care’ defined as not missing scheduled follow-up date, but not yet started on ART; 2) started on ART; 3) LTFU, defined as not coming for follow-up visit within the 90 days after the last scheduled appointment; and 4) death. Patients having outcomes of LTFUs and deaths were combined together when estimating ‘attrition’ as primary outcome measure because many of LTFUs might be unascertained deaths (12).

### Statistical analysis

STATA (version 12.1 STATA Corp., College Station, TX, USA) was used for data analysis. The baseline demographic, clinical, and immunological characteristics of patients were analysed. Survival analysis was undertaken to analyse the hazards of attrition after enrolment. The risk time of patients in pre-ART care started on their date of enrolment. For patients who developed attrition events (i.e. death or LTFU), the last scheduled appointment date (i.e. patient was scheduled to come but did not turn up) was the date of failure event. The last scheduled appointment date (up to 31^st^ December 2015) was the date of censoring for patients who were retained in follow-up. Patients who were initiated on ART were censored on the date of ART initiation. Overall and stratum-specific cumulative attrition rates (death rates and LTFU rates) were estimated and expressed as attrition per 100 person-years (PYs). The cumulative incidence of attrition at monthly intervals for the first 6 months and then yearly from year 1 onwards was calculated as the proportion of patients in the cohort who had undergone attrition by the end of each period of follow-up. Associations between demographic, clinical, and immunological characteristics and attrition were studied, after excluding patients with missing values. Log-rank tests and bivariate unadjusted hazard ratios (HRs) were calculated. Characteristics found statistically significant during bivariate analysis (*p*<0.2) were entered into a Cox proportional hazard regression model, and adjusted HRs were calculated. Model selection was based on comparisons of change in model-2 log-likelihood in backward stepwise manner. Interaction between WHO Stage and CD4 counts was examined. Proportionality assumptions were tested using Schoenfeld residuals, log–log plots, and observed versus predicted survival plots. Kaplan–Meier survival curves (showing the cumulative incidence of attrition) across strata of key variables associated with attrition were plotted. Statistical significance was assessed at 5% probability of type-1 error and 95% confidence intervals (95% CI) were estimated for attrition rates and HRs.

## Results

**Fig. 2:**
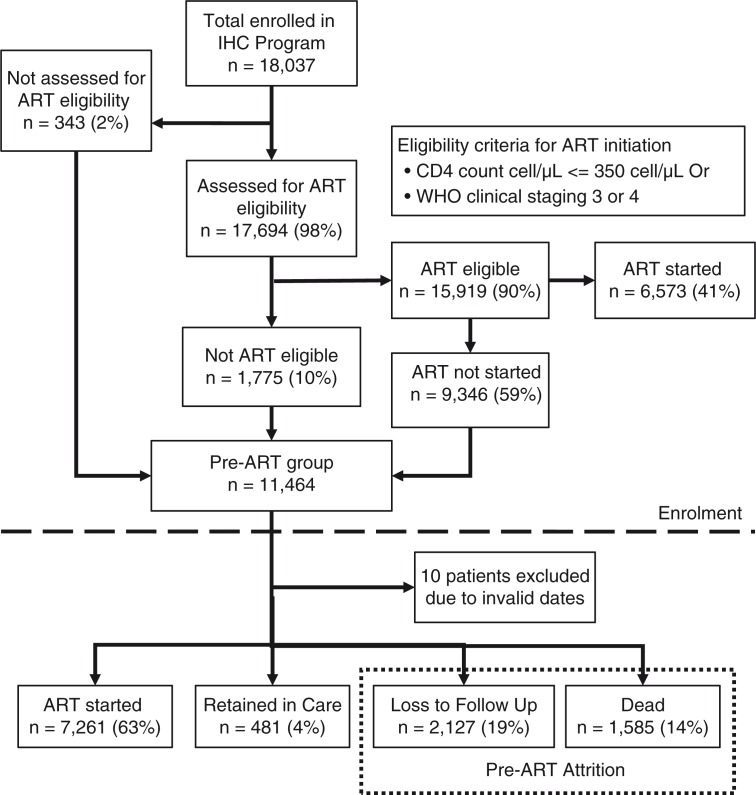
Flowchart showing HIV-infected adults entering pre-ART care in the Integrated HIV Care programme, Myanmar, 2011–2014.

In total, 18,037 HIV-infected adults were enrolled during the reference period. Of these patients, 17,694 (98%) were assessed for ART eligibility and 15,919 (90%) were found ART eligible. Among the ART eligible, only 6,573 (41%) were started on ART at enrolment. Thus, a total of 11,464 patients entered into pre-ART care group (Fig. 2). Of these, 10 patients had invalid dates and were removed from our analysis.

The demographic, clinical, and immunological characteristics of the study group are shown in Table 1. About 60% were men, and the mean age (standard deviation) was 37 (10) years. Literacy levels were high at 88% (10,112), whereas 25% (2,881) participants were unemployed. Only 6,521 (57%) of participants were married with another 19% being single and 23% either widowed or separated. Nearly half were in WHO Stage 3 and median CD4 count was 160 cells/µL (interquartile range: 69–312); 874 (8%) patients had CD4 count more than 500 cells/µL. Nearly half had BMI of less than 18.5 kg/m^2^ and two-thirds were anaemic. The proportion of missing records was high for CD4 count (15%), BMI (21%), and haemoglobin level (16%) (Table 1).

**Table 1:** Demographic, clinical and immunological characteristics of HIV-infected adults who entered pre-ART care of the Integrated HIV Care (IHC) programme, Myanmar, 2011–2014

Patient characteristics		Number (%)
Sex	Female	4,848 (42)
	Male	6,606 (58)
Age (years)	16–25	699 (6)
	25–34	4,269 (37)
	35–44	4,378 (38)
	≥45	2,108 (18)
Marital status	Single	2,123 (19)
	Married	6,521 (57)
	Widowed	1,940 (17)
	Divorced/separated	708 (6)
	Unknown	162 (1)
Literate	No	1,198 (10)
	Yes	10,112 (88)
	Unknown	144 (1)
Employed	No	2,881 (25)
	Yes	8,228 (72)
	Unknown	345 (3)
WHO Stage^a^	Stage 1	2,609 (23)
	Stage 2	1,968 (17)
	Stage 3	5,220 (46)
	Stage 4	1,519 (13)
	Unknown	138 (1)
CD4 count (cells/µL)^a^	< 50	1,687 (15)
	50–99	1,670 (15)
	100–199	2,285 (20)
	200–349	2,094 (18)
	350–499	1,149 (10)
	>500	874 (8)
	Unknown	1,695 (15)
BMI (kg/m^2^)^a^	<18.5	3,871 (34)
	18.5–25	4,262 (37)
	25–30	618 (5)
	30–35	142 (1)
	>35	118 (1)
	Unknown	2,443 (21)
Haemoglobin level^a^	Normal	3,220 (28)
	Mild anaemia	2,184 (19)
	Moderate anaemia	3,285 (29)
	Severe anaemia	951 (8)
	Unknown	1,814 (16)
Hepatitis C infection^a^	No	8,255 (72)
	Yes	769 (7)
	Unknown	2,430 (21)
Hepatitis B infection^a^	No	8,268 (72)
	Yes	751 (7)
	Unknown	2,435 (21)

a Measured at enrolment; HIV, human immunodeficiency virus; ART, antiretroviral therapy; 100 PYs, 100 person-years of observation; CI, confidence interval; WHO, World Health Organization; BMI, body mass index; Hb, haemoglobin.

The pre-ART group had a total observation period of 4,788 PYs, during which 3,712 attrition events occurred: 1,585 (43%) deaths and 2,127 (57%) LTFU. The overall attrition rate was 78 per 100 PYs (95% CI, 75–80). Based on the nature of entry in the pre-ART cohort, the attrition rate among 343 patients not assessed for ART eligibility was 92 (95% CI, 77–110), 1,776 patients assessed for ART but not found eligible was 42 (95% CI, 39–45), and the 9,346 patients found ART eligible but not started was 98 (95% CI, 94–101) per 100 PYs. Of the patients entering pre-ART care, 7,261 (64.4%) were subsequently initiated on ART at a median follow-up time interval of 77 days.

The cumulative incidence of attrition and the nature of attrition are shown in Table 2. It was observed that 25% of the pre-ART cohort underwent attrition within the first 3 months. Nearly 90% of all attrition events occurred during the first 6 months of enrolment. Among attrition within the first month, the proportion due to deaths and LTFU were 40 and 60%, which changed to 51 and 49%, respectively, by the end of second month. The contribution of deaths to total attrition was high initially and decreased over time.

**Table 2:** Cumulative incidence of attrition (in %) and nature of attrition among HIV-infected adults who entered pre-ART care of the Integrated HIV Care programme, Myanmar, 2011–2014

	Attrition				
					Cumulative incidence
Time interval after enrolment in pre-ART care	Total numbers	Deaths (% within attrition)	LTFUs (% within attrition)	Censored Patients (ART initiated, RFU)	Attrition (%) at end of interval (95% CI)^a^
0–1 month	1,602	638 (39.8)	964 (60.2)	–	14 (13–15)
1–2 months	629	321 (51)	308 (49)	2,099	20 (19–21)
2–3 months	382	202 (52.9)	180 (47.1)	2,251	25 (24–26)
3–4 months	321	119 (37.1)	202 (62.9)	851	31 (30–32)
4–5 months	165	92 (55.8)	73 (44.2)	424	35 (34–36)
5–6 months	104	50 (48.1)	54 (51.9)	300	37 (36–39)
0.5–1 year	266	93 (35)	173 (65)	864	46 (45–48)
1–2 years	170	46 (27.1)	124 (72.9)	574	56 (54–58)
2–3 years	57	13 (22.8)	44 (77.2)	249	64 (62–66)
3–4 years	16	11 (68.8)	5 (31.3)	111	70 (67–74)
>4 years	–	–	–	19	70 (67–74)

a Cumulative failure till the end of specified interval; HIV, human immunodeficiency virus; ART, antiretroviral therapy; LTFU, loss-to-follow-up; RFU: retained in follow-up.

**Fig. 3:**
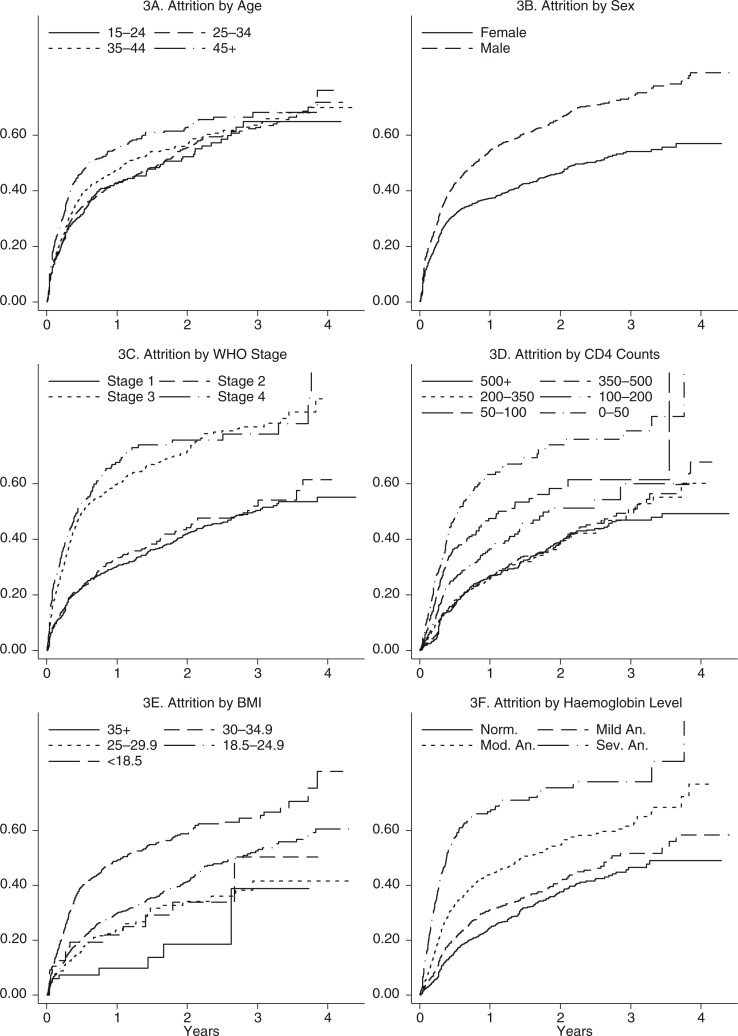
Cumulative incidence of attrition by age, sex, WHO Stage, CD4 counts, BMI and haemoglobin levels among patients who entered into pre-ART care, Myanmar, 2011–2014. WHO, World Health Organization; BMI, body mass index; ART, antiretroviral therapy; Norm., normal; Mod., moderate; Sev., severe; An., anaemia.

Stratum-specific attrition rates by patient characteristics and the estimated HRs for attrition are shown in Table 3. The highest attrition rates were observed among patients in WHO Stage 3 and 4, and among the severely anaemic. On bivariate analysis, the hazard of attrition was higher among patients who were male, aged >45 years, not married, illiterate, in WHO clinical Stage 3 or 4, had a CD4 count of less than 200 cell/µL, had BMI <25 kg/m^2^, and had anaemia (Table 3). In a multivariate Cox proportional hazards model, being male, in WHO Stage 3, having a CD4 count less than 50 cell/µL, having a BMI<25 kg/m^2^, and having moderate or severe anaemia, were factors independently associated with a higher risk of attrition. However, because the covariates, apart from the WHO staging, were found to violate the proportionality assumptions, the adjusted analysis results are not presented here. The unadjusted cumulative incidence of attrition by age, sex, WHO Stage, CD4 counts, BMI, and haemoglobin levels over the period of follow-up is presented in Fig. 3. Interaction between WHO Stage and the CD4 count was not statistically significant.

**Table 3:** Factors associated with attrition among HIV-infected adults who entered into pre-ART care in IHC programme, Myanmar, 2011–2014

Patient characteristics	Attrition	Attrition rate per 100 person-years (95% CI)	Unadjusted hazard ratio (95% CI)
Sex			
Female	1,344	56.3 (53.4–59.4)	1
Male	2,368	98.7 (94.8–102.8)	1.5 (1.4–1.6) a
Age (years)			
15–24	251	55.6 (49.2–63)	1
25–34	1,348	65.7 (62.3–69.3)	1 (0.9–1.2)
35–44	1,337	83.4 (79–87.9)	1.1 (1.0–1.3)
≥45	776	114.1 (106.3–122.4)	1.5 (1.3–1.7) a
Marital status			
Single	758	97.5 (90.8–104.7)	1.3 (1.2–1.4) a
Married	1,996	66.9 (64.1–69.9)	1
Widowed	616	83.6 (77.2–90.5)	1.1 (1.0–1.2) a
Divorced/separated	257	109.3 (96.7–123.5)	1.4 (1.2–1.6) a
Literate			
No	509	103.9 (95.3–113.4)	1.5 (1.3–1.6) a
Yes	3,119	73.3 (70.8–75.9)	1
Employed			
No	993	75.7 (71.1–80.5)	1
Yes	2,555	77.4 (74.5–80.5)	1.1 (1–1.1) a
WHO Stage^b^			
Stage 1	698	35.1 (32.6–37.8)	1
Stage 2	418	45.2 (41.1–49.8)	1.1 (0.9–1.2)
Stage 3	1,885	126.2 (120.6–132)	2.4 (2.2–2.7) a
Stage 4	597	165.5 (152.7–179.3)	3.0 (2.7–3.3) a
CD4 count (cells/µL)^b^			
<50	518	113.3 (104–123.5)	3.4 (2.9–3.9) a
50–99	368	77.4 (69.8–85.7)	2.3 (2–2.8) a
100–199	353	50.8 (45.7–56.4)	1.5 (1.3–1.8) a
200–349	296	33.4 (29.8–37.4)	1.1 (0.9–1.3)
350–499	281	28.5 (25.4–32.1)	1.1 (0.9–1.2)
>500	266	24.8 (22–28)	1
BMI (kg/m^2^)^b^			
<18.5	1,117	82.2 (77.5–87.2)	4.1 (2.4–7.1) a
18.5–25	839	37.5 (35–40.1)	2.2 (1.3–3.7) a
25–30	100	28.2 (23.2–34.3)	1.7 (1.0–3.1)
30–35	26	33.7 (23–49.5)	2.1 (1.1–4.0) a
>35	13	14.7 (8.5–25.3)	1
Haemoglobin level^b^			
Normal	471	26 (23.7–28.4)	1
Mild anaemia	383	35 (31.7–38.7)	1.3 (1.2–1.5) a
Moderate anaemia	801	62.1 (58–66.6)	2.2 (2.0–2.5) a
Severe anaemia	403	144.3 (130.9–159.1)	4.8 (4.2–5.5) a
Hepatitis C infection^b^			
No	1,737	46.8 (44.6–49)	1
Yes	186	52.9 (45.8–61)	1.1 (1.0–1.3)
Hepatitis B infection^b^			
No	1,764	46.8 (44.6–49)	1
Yes	156	52.9 (45.2–61.9)	1.1 (0.9–1.3)

a *p*-value<0.05

b measured at enrolment; ‡ The final adjusted Cox-model was tried and included the covariates sex, age, WHO Stage, CD4 count, BMI, and haemoglobin levels. The results are not presented here as all covariates variables were found to violate proportionality assumptions.

HIV, human immunodeficiency virus; ART, antiretroviral therapy; HR, hazard ratio; BMI, body mass index; WHO, World Health Organization

## Discussion

This is the first study from Myanmar on pre-ART attrition among HIV-infected patients. Pre-ART patients are one of the most neglected subgroups of PLHIV. Attrition rates among pre-ART groups worldwide are higher as compared to those receiving ART (5, 6, 13, 14). In Myanmar, within the IHC programme, an alarmingly high attrition rate was observed with nearly 70% being either dead or LTFU at the end of 4 years. This is one of the highest rates of attrition ever reported, in line with published reports from Asia and Africa (6–8, 15–21). Most of the attrition occurred in the early stages of enrolment into care: 90% of attrition occurred within first 6 months with nearly half in the first month. This is worrisome and requires urgent attention. Although risk of attrition was higher among those with low CD4 counts and in WHO Stage 3 or 4, males, patients with low BMI, or those who are unmarried, attrition rates remained high across all strata. These findings are similar to those reported in previous studies (7, 8, 17, 18).

The median CD4 count was low at 160 cells/µL indicating advanced disease at presentation, delays in care seeking by PLHIV, and delayed diagnosis. This is possibly a reason for high dead cases during the first month. To address this, HIV testing should be made more widely available in the country. Currently, national guidelines in Myanmar recommend HIV testing of high-risk groups, pregnant women, new military recruits, and TB patients (4, 22). Efforts should be made to increase HIV test coverage in these groups. Another group to consider in regard to offering HIV testing is ‘presumptive TB patients’. Several studies have shown that HIV prevalence in this group is as high as that among TB patients suggesting the need for screening and early diagnosis of HIV (23).

Even among those found to be eligible for ART, more than half started on ART only 6 weeks after enrolment, suggesting delayed ART initiation. This might have contributed to the high mortality found in our study which is also in line with previous studies (24–27). The attrition rate in this group (eligible for ART) was the highest, when compared with those who were not assessed and those who were assessed but not eligible for starting ART.

It appears that starting a patient on ART is the best way of retaining patients in care (28, 29). We recommend starting all PLHIV diagnosed on ART irrespective of CD4 count or clinical stage in line with the recent WHO recommendation of ‘test and treat’ approach (30). It is encouraging to note that Myanmar NAP has already adopted the WHO-2013 guideline (CD4 threshold of 500) since March 2015 (10, 22). With this, the proportion on pre-ART care will reduce substantially. Given this preparedness and because only 8% of PLHIV have CD4 counts of more than 500 thereby requiring ART, it might be feasible to ‘test and treat’ all with little additional burden on the programme.

Until the country moves into ‘test and treat’ strategy, we need to ensure that patients placed on pre-ART care are monitored well. We recommend introducing an indicator in the monthly report for monitoring retention and/or attrition among pre-ART cohort (31) because current reports in HIV care mostly focus on those receiving ART. Having an indicator showing how many patients are lost or died before starting on ART can give a clear view of how well we are doing before giving them ART. The care package currently provided (treatment for opportunistic infection, multivitamins, and counselling service) to pre-ART group needs to be strengthened by including provision of nutritional support and needs serious consideration because attrition is linked to low BMI and low haemoglobin. Currently, a number of patient visits (at least two and sometimes three to four) are required for full assessment and clinical decision-making. Steps, such as point-of-care CD4 testing and automated analyzers (32), have the potential to expedite assessment procedures and reduce the number of patient visits and the attrition that occurs during the process (6). Finally, we need to ensure that the systematic patient tracing systems are utilized for all patients and not limited only to those on ART. The systematic tracing system that is currently used for patients on ART who miss an appointed date includes tracing these patients every 2 weeks for 2 months and then monthly for 3 months until they come back or for a maximum of 7 months if they do not come back, using mobile phones or home visits by PLHIV volunteers.

### Strengths

This study has several strengths. Firstly, the information is drawn from the second largest cohort of PLHIV in Myanmar with long follow-up data providing reliable estimates of attrition. Secondly, the data come from programmatic settings, thus reflecting the realities on the ground. Thirdly, this fills a knowledge gap in Myanmar about attrition among pre-ART care. Although there are many studies of ART cohorts, no previous study has examined pre-ART cohort in Myanmar (5). Fourthly, we used robust methods of data cleaning and analysis including multivariate survival analysis to examine factors associated with attrition. Finally, we followed the strengthening the reporting of observational studies in epidemiology (STROBE) guidelines for conducting and reporting this research in a scientific and ethical manner (33, 34).

### Limitations

As always, there are some limitations and they are mainly related to the operational nature of this study. Firstly, we used a definition of 90 days after the last appointed date to define LTFU. Given that the visits in pre-ART care were scheduled to be between 3 and 6 months apart for clinically healthy patients, we had to wait for 6 to 9 months before declaring LTFU. Hence, it is possible that some of the patients categorized as ‘retention in pre-ART’ at the time of assessment could actually be LTFU or dead. Thus, we could be underestimating our attrition rates. On the other hand, it is possible that many of those identified as having dropped out could be receiving HIV care in other facilities, without our knowledge (6). Because there is no systematic tracking of the patients in pre-ART care, we do not know the magnitude of this crossover (6). LTFU contributed to more than half of this overall rate. As half of LTFU patients were actually reported as dead in one study (12), one could make an assumption that in fact, death rate might be higher. Secondly, substantial proportions of missing data were found on some important variables such as CD4 count, body mass index, and haemoglobin levels, which were all measured at enrolment. A missing data analysis revealed that males were more likely to have missing data as compared to females. Younger participants too were more likely to have missing hepatitis B and hepatitis C results, whereas older participants are likely to have missing BMI and CD4 results. This is likely because of higher rates or early attrition among males. This could also be related to problems in documentation, which currently is mostly paper-based. A possible solution might be to institute a real-time web-based management information system with data entry at each clinic and laboratory. Thirdly, as the nature of paediatric or pregnant women under the PMTCT cohort and adult cohort are different, we cannot apply the findings of this study to these cohorts. Lastly, we did not explore the actual reasons behind attrition among pre-ART group in this study. A better understanding of these reasons warrants further research, especially using qualitative methods with in-depth interviews of patients, family members, and providers.

## Conclusion

Attrition in a pre-ART HIV care cohort in Myanmar was alarmingly high – with most occurring within the first 6 months. Strategies such as improving early HIV diagnosis and the early initiation of ART, providing comprehensive nutrition support, implementing intensive monitoring using newly developed indicators, and tracking patients who do not return for their appointments are needed. It is most important that Myanmar moves towards universal ART and eliminate the need for pre-ART care.

## Open-access statement

In accordance with WHO's open-access publication policy for all work funded by WHO or authored/co-authored by WHO staff members, the WHO retains the copyright of this publication through a Creative Commons Attribution IGO license (www.creative commons.org/licenses/by/3.0/igo/legalcode), which permits unrestricted use, distribution, and reproduction in any medium provided the original work is properly cited.

## Paper context

High retention rates have been documented among patients receiving antiretroviral therapy (ART) in Myanmar. However, there is no information on HIV-infected individuals in pre-ART care making it is the most neglected part of the HIV care chain. In this study, alarmingly high attrition (death or lost-to-follow-up) rates in pre-ART care were observed, especially within the first six months after registering for care. These results lend support to a strategy of ‘Test and Treat’ for all PLHIV in Myanmar.
